# Stability of symptom networks for depression, anxiety, and PTSD among psychiatric patients during the COVID-19 pandemic: A case-control study using network temperature

**DOI:** 10.1192/j.eurpsy.2026.11022

**Published:** 2026-07-31

**Authors:** A. K. C. Chau, J. K. N. Chan, C. S. M. Wong, R. S. T. Chu, W. C. Chang

**Affiliations:** 1 Department of Psychiatry; 2 School of Public Health, The University of Hong Kong, Hong Kong, Hong Kong

## Abstract

**Introduction:**

The network theory of psychopathology posits that psychiatric disorders emerge from dynamic interactions among underlying symptoms. These interactions can lead to two alternative stable states: an ‘all-on’ state, where all symptoms are activated as in a full-blown mental disorder, and an ‘all-off’ state, where all activations dissipate, resulting in a healthy state. Network temperature (NT) has been proposed as a metric to measure the stability of symptom network, with a lower NT indicating a more ordered network in which symptoms are more aligned with either of the stable states. Limited research has used NT to understand the stability of symptom networks in psychiatric populations. In particular, the COVID-19 pandemic offers a unique opportunity to examine this, as elevated symptoms in psychiatric patients compared to healthy controls (HC) may reflect distinct network dynamics.

**Objectives:**

We compared the NT of symptom network of depression, anxiety and post-traumatic stress disorder (PTSD) between psychiatric patients and HC recruited during the 5^th^ wave of the COVID-19 pandemic in Hong Kong.

**Methods:**

Treatment-receiving psychiatric outpatients (N= 415) and demographically-matched HC (N= 399) completed the Patient Health Questionnaire-9, the Generalized Anxiety Disorder Scale-7, and the Impact of Event Scale in March–April 2020 to measure their depressive, anxiety, and PTSD symptoms, respectively. Networks of these symptoms were estimated separately using multigroup Ising models, with NT in HC fixed at 1. The bootstrapped 95% confidence intervals (CI) for estimates of NT were computed using 1000 group-stratified bootstrap samples. We compared NT of these symptoms between patients and HC, and then among patients with affective disorders (AD, n= 229), patients with psychotic disorders (PD, n= 138) and HC.

**Results:**

Patients had more severe and varied depressive, anxiety and PTSD symptoms than HC. Compared with HC, patients had lower NT for anxiety (0.85, 95% CI: 0.77-0.94) and PTSD (0.87, 95% CI: 0.78-0.97), but not depression. Subgroup analyses suggested that patients with AD (but not PD) exhibited lower NT for depression (0.90, 95% CI: 0.75-0.95) and anxiety (0.83, 95% CI: 0.75-0.95) than HC. The NT of PTSD was marginally lower in patients with AD (0.89, 95% CI: 0.79-1.00) and PD (0.85, 95% CI: 0.76-1.00) than in HC.

**Conclusions:**

Compared with HC, psychiatric patients exhibited lower NT for anxiety and PTSD symptoms during the COVID-19 pandemic, indicating reduced stability in these symptom networks that potentially remained predominantly in the ‘all-on’ state. The increased polarization of psychiatric symptoms was more prominent in patients with AD. Our results highlight the utility of NT in understanding the vulnerability of psychiatric populations during the COVID-19 pandemic from a dynamic network perspective.

**Disclosure of Interest:**

None Declared

